# Universality of indeterminate growth in lizards rejected: the micro-CT reveals contrasting timing of growth cartilage persistence in iguanas, agamas, and chameleons

**DOI:** 10.1038/s41598-019-54573-5

**Published:** 2019-12-12

**Authors:** Petra Frýdlová, Jana Mrzílková, Martin Šeremeta, Jan Křemen, Jan Dudák, Jan Žemlička, Pavel Němec, Petr Velenský, Jiří Moravec, Daniel Koleška, Veronika Zahradníčková, Tomáš Jirásek, Petr Kodym, Daniel Frynta, Petr Zach

**Affiliations:** 1 Department of Zoology, Faculty of Science, Charles University, Viničná 7, CZ-12844 Prague, Czech Republic; 2 Specialized laboratory of experimental imaging, Ruská 2411/87, CZ-10000 Prague, Czech Republic; 3 Department of Anatomy, Third Faculty of Medicine, Charles University, Ruská 2411/87, CZ-10000 Prague, Czech Republic; 4 Institute of Experimental and Applied Physics, Czech Technical University in Prague, Husova 5, CZ-11000 Prague, Czech Republic; 5 Prague Zoo, U Trojského Zámku 3, CZ-17100 Prague, Czech Republic; 6 Department of Zoology, National Museum, Cirkusová 1740, CZ-19300 Prague, Czech Republic; 7 Department of Zoology and Fisheries, Faculty of Agrobiology, Food and Natural Resources, Czech University of Life Sciences Prague, Kamýcká 129, CZ-16500 Prague, Czech Republic; 8 Zoological and Botanical Garden Pilsen, Pod Vinicemi 9, CZ-30116 Pilsen, Czech Republic; 9 National Institute of Public Health, Šrobárova 48, CZ-10042 Prague, Czech Republic

**Keywords:** Evolutionary developmental biology, Herpetology

## Abstract

Squamate reptiles are considered to exhibit indeterminate growth. Nevertheless, current literature disputes the available definitions of this growth type, presents new theoretical models, and questions its universality in cold-blooded vertebrates. We have followed up on our previous research employing micro-CT to explore growth plate cartilage (GPC) in the epiphysis of long bones, which is responsible for longitudinal skeletal growth by the endochondral ossification process. We focused on numerous and highly diversified group of the Iguania clade comprising Acrodonta (agamas and chameleons) and Pleurodonta (“iguanas”). We recorded the absence of GPC in most of the examined adult Pleurodonta specimens and interpret it as an irreversible arrest of skeletal growth. This finding clearly rejects the universality of indeterminate growth in lizards. On the other hand, we found apparent GPC preservation in most of the adult specimens belonging to Acrodonta. This suggests a preserved ability to continue body growth throughout most of their life. We discuss the uncovered disparity between Acrodonta and Pleurodonta and emphasize the importance of GPC degradation timing.

## Introduction

Postnatal skeletal growth connected with the increase in skeletal size is traditionally divided into the determinate and indeterminate type^1^. The main difference is in the ability to continue growth throughout the life in indeterminate growers, while the determinate ones cease their skeletal growth typically close to sexual maturation^2^. Sebens^3^ brought more accurate definitions with a detailed description of growth curves which are variations on attenuating or asymptotic growth. Ectothermic vertebrates (fish, amphibians and reptiles) are considered as groups with indeterminate body growth^4–9^ (but see^10–16^), while endotherms (birds and mammals) are determinate growers^4,17^ (but see^18–21^). Nevertheless, current literature is pointing to the problematic classification of animal taxa to specific groups according to the available definitions of body growth^22–25^.

In our previous comparative study, we employed advanced imaging methods (micro-radiography and micro-computed tomography) to evaluate growth abilities in monitor lizards (Varanidae) according to the presence/absence of the growth plate cartilage (GPC) in the epiphysis of long bones^22^. In fully grown specimens of small-bodied species, we clearly demonstrated the degradation of GPC. It is a sign of determinate growth as it arrests the growth irreversibly (for the description of the cellular process of growth plate degradation see^26–32^ and references therein). In large-bodied species of monitor lizards, we found a contrasting growth pattern typical for indeterminate growers. Adults, except for very old senescent individuals, retained GPC nearly throughout their entire life. We interpreted this dual pattern of body growth in monitor lizards as an extreme case of heterochrony^22^.

**Figure 1:**
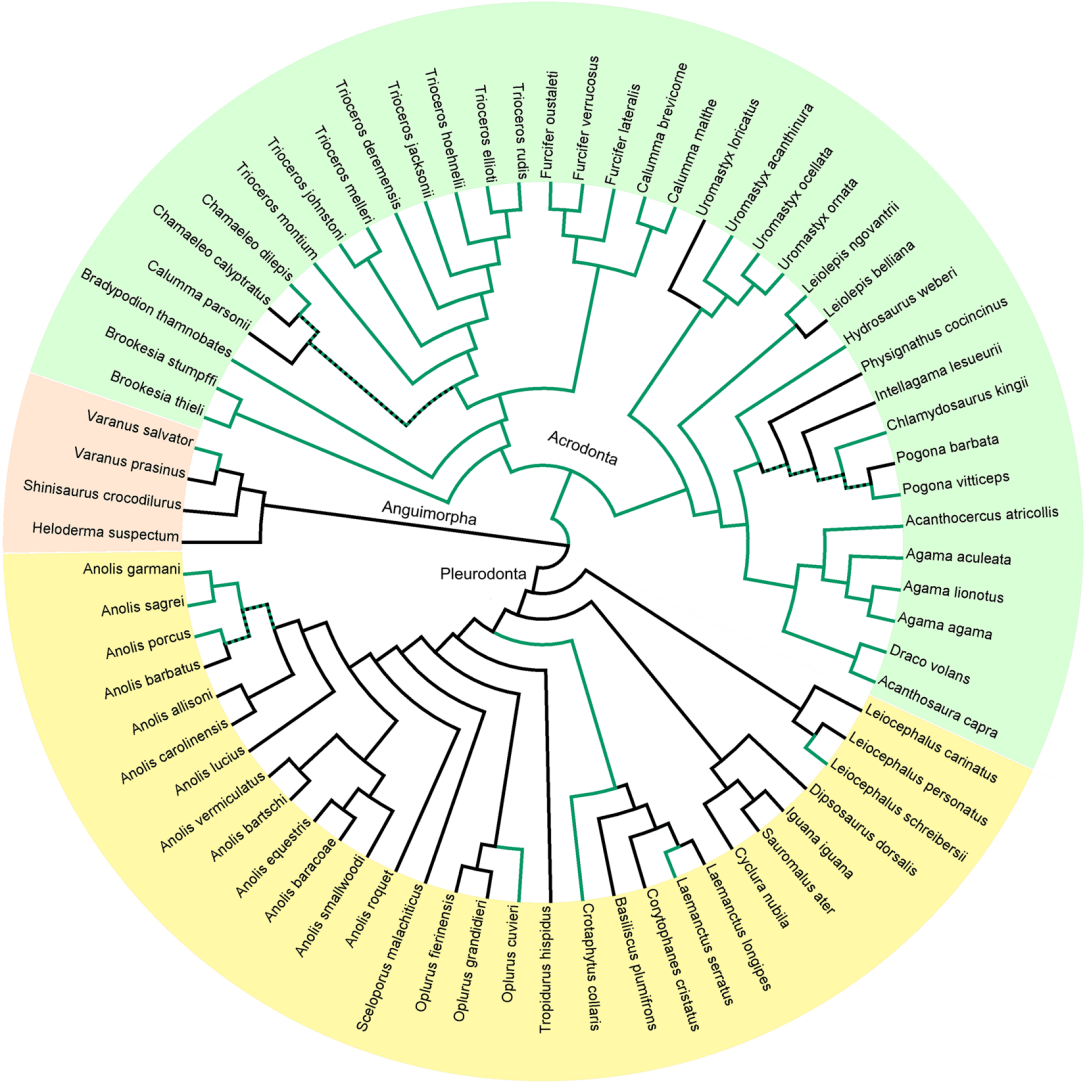
Phylogenetic pattern of the growth plate cartilage (GPC) across Iguania. Visualization of GPC presence (green) and absence (black) within the Iguania clade comprising Acrodonta (agamas and chameleons) and Pleurodonta (“iguanas”). Reconstruction of ancestral states was done using the parsimony method implemented in Mesquite (Maddison and Maddison 2015). Monitor lizards (Varanidae), the beaded lizard (Helodermatidae) and Chinese crocodile lizard (Shinisauridae) were used as outgroup (Anguimorpha).

The dual pattern of body growth in monitor lizards violates the universality of indeterminate growth in squamate reptiles. Nevertheless, the reported association of the growth pattern with the adult body size of the species may be attributed to an extreme evolution of body size in this otherwise homogeneous group of lizards. Thus, we searched for other clades of squamates with great variation in body size but exhibiting contrasting life strategies. We focused on Iguania (sensu^33^) comprising the subclades of Acrodonta (chameleons and agamas) and Pleurodonta (Iguanidae and related families, hereafter also called “iguanas”). Those two crown subclades of lizards (for phylogenetic relationships see Fig. 1) have undergone convergent evolution. They feature unique evolutionary history and specific ecological strategies, which makes it possible to search for the putative selective pressures involved in the regulation of body growth and its possible arresting. The goals of this study were (1) to employ advanced imaging techniques to evaluate the presence/absence of GPC in examined specimens; (2) to score the species according to the pattern of GPC persistence in adults; (3) to test the universality of the presumed indeterminate growth in this clade, and (4) to discuss the putative life-history parameters responsible for the body growth pattern.

## Results

**Figure 2:**
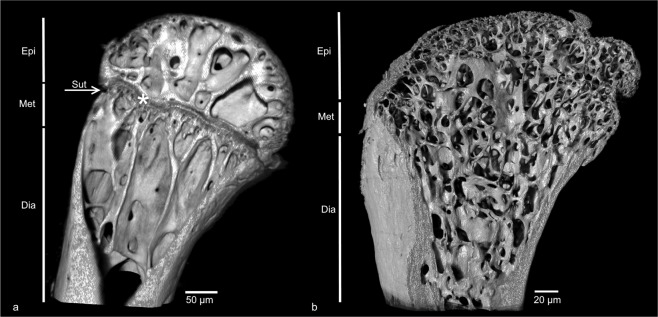
Visualisation of proximal part of the femur by micro-CT. Frontal cross-section of the proximal part of the femur. The epiphyseal growth plate is present in adult *Uromastyx ornatus* (**a**) and completely absent in adult old *Chamaeleo calyptratus*. (**b**) Abbreviations: Epiphysis (Epi), Metaphysis (Met), Diaphysis (Dia), Suture (Sut), Epiphyseal growth plate (asterisk).

We analysed 150 bones of agamid (38), chamaeleonid (46) and “iguanid” (67) lizards to determine the presence/absence of epiphyseal growth plates in the femoral epiphysis. We confirmed that the employment of micro-radiography and micro-CT enables detailed visualization of epiphyseal growth plates (presence/absence/the process of degradation; Figs. 2, SI1). The presence of the growth plate cartilage was scored in the following cases: (1) epiphysis and diaphysis separated by a wide radio-translucent band corresponding to non-calcified growth cartilage in the area near metaphysis; (2) the trabeculae not extended into the metaphysis; and (3) the suture between the epiphysis and the metaphysis was present (Figs. 2a, SI2, SI3). The absence of the growth plate cartilage was scored when: (1) the radio-translucent band corresponding to non-calcified growth cartilage was absent, which is a sign of growth plate senescence, degradation or even its complete absence; (2) the suture between the epiphysis and the metaphysis was not present; and (3) the whole proximal part of the femur was occupied by a continuous network of bone trabeculae (Figs. 2b, SI4, SI5). We detected the process of GPC degradation as well (labelled with ± in Table 1). In that case, (1) the radio-translucent band corresponding to non-calcified growth cartilage was not present in whole cross-section of femoral epiphysis, but just partly; (2) the suture between the epiphysis and the metaphysis was present only on one side of the femoral epiphysis or was absent; (3) the area of primary and secondary ossification centres was mostly occupied by a network of bone trabeculae and partly encroached to metaphysis (SI1, SI6, SI7). For formal analyses, we used binary data concerning the GPC state (presence/absence). The results of micro-CT examinations and data on age, sex and body size of the studied specimens are summarized in Table 1 (for references concerning SVL_max_ see SI8).

**Table 1:** Epiphyseal state in the proximal epiphysis of the femur in the examined species of the Iguania clade.

Family	Species	GPC	SVL	SVL_rel_	Sex	Age	Source
Agamidae	*Acanthocercus atricollis*	+	131.5	78.7	*M*	A	CUNI
	*Acanthosaura capra*	+	132.1	95.8	*M*	A	CUNI
	*Agama aculeata*	+	89.7	76.7	*M*	A	CUNI
	*Agama agama*	+	138.0	100.7	*M*	A	CUNI
	*Agama lionotus dodomae*	+	138.0	101.0	*M*	A	Z. Prague
	*Agama lionotus dodomae*	+	140.0	102.4	*M*	A	Z. Prague
	*Agama lionotus dodomae*	+	115.0	84.1	*F*	A	Z. Prague
	*Agama lionotus dodomae*	+	125.0	91.4	*F*	A	Z. Prague
	*Agama lionotus dodomae*	+	105.0	76.8	*F*	A	Z. Prague
	*Agama lionotus dodomae*	±	129.0	94.4	*F*	A	Z. Prague
	*Agama somalica*	+	70.0	68.0	*F*	A	CUNI
	*Draco volans*	+	72.1	84.8	*M*	A	CUNI
	*Hydrosaurus weberi*	+	279.8	84.8	*M*	A	CUNI
	*Chlamydosaurus kingii*	+	234.0	92.1	*M*	A	CUNI
	*Intellagama lesueurii*	−	166.0	54.6	*M*	A	NMP
	*Leiolepis belliana*	−	135.0	75.9	*F*	A	CUNI
	*Leiolepis ngovantrii*	+	104.6	82.7	*F*	A	CUNI
	*Paralaudakia caucasia*	+	113.0	65.3	*M*	A	CUNI
	*Physignathus cocincinus*	−	165.0	82.5	*F*	A	Z. Prague
	*Physignathus cocincinus*	−	222.0	88.8	*M*	A	NMP
	*Physignathus cocincinus*	−	227.0	113.5	*F*	A	CUNI
	*Physignathus cocincinus*	+	178.0	71.2	*M*	A	CUNI
	*Pogona barbata*	−	215.0	86.4	*F*	A	CUNI
	*Pogona vitticeps*	+	211.0	97.7	*M*	A	CUNI
	*Saara loricata*	−	285.0	98.3	*M*	A (>30)	CUNI
	*Saara loricata*	−	255.0	87.9	*M*	A (28)	CUNI
	*Uromastyx acanthinura*	+	180.0	71.1	*F*	A	Z. Pilsen
	*Uromastyx acanthinura*	+	189.0	74.7	*M*	A	Z. Prague
	*Uromastyx acanthinura*	+	105.0	41.5	*F*	SA	Z. Prague
	*Uromastyx acanthinura*	+	120.0	47.4	*M*	SA	Z. Prague
	*Uromastyx acanthinura*	+	71.0	28.1	*M*	SA	Z. Prague
	*Uromastyx aegyptia*	+	250.0	66.7	*M*	A (5)	CUNI
	*Uromastyx ocellata*	+	173.0	99.4	*F*	A	Z. Dubeč
	*Uromastyx ocellata*	+	168.0	96.6	*M*	A	Z. Dubeč
	*Uromastyx ornata*	±	177.0	107.3	*M*	A	Z. Ústí
	*Uromastyx ornata*	+	150.0	76.5	*F*	A	Z. Ústí
	*Uromastyx ornata*	+	140.0	71.4	*M*	A	Z. Ústí
	*Uromastyx ornata*	+	184.8	94.3	*F*	A	CUNI
Corytophanidae	*Basiliscus plumifrons*	−	170.6	101.5	*M*	A	CUNI
	*Corytophanes cristatus*	−	110.8	88.6	*F*	A	CUNI
	*Laemanctus longipes*	−	133.0	95.0	*F*	A	Z. Pilsen
	*Laemanctus longipes*	−	112.0	80.0	*F*	A	Z. Pilsen
	*Laemanctus longipes*	−	129.0	92.1	*F*	A (12)	CUNI
	*Laemanctus serratus*	+	85	85	*M*	A	CUNI
Crotaphytidae	*Crotaphytus collaris*	±	85.0	78.6	*M*	A	Z. Dubeč
	*Crotaphytus collaris*	+	76.0	70.2	*M*	A	CUNI
	*Crotaphytus collaris*	+	85.0	89.6	*F*	A	CUNI
Dactyloidae	*Anolis allisoni*	−	55.0	73.3	*F*	A	CUNI
	*Anolis allisoni*	−	60.0	80.0	*F*	A	CUNI
	*Anolis allisoni*	−	80.0	80.0	*M*	A	CUNI
	*Anolis allisoni*	−	74.0	74.0	*M*	A	CUNI
	*Anolis baracoae*	+	116.0	74.8	*F*	A	CUNI
	*Anolis baracoae*	+	146.0	84.9	*M*	A	Z. Dubeč
	*Anolis baracoae*	−	141.0	91.0	*F*	A	CUNI
	*Anolis baracoae*	−	133.0	85.8	*F*	A	CUNI
	*Anolis barbatus*	+	123.0	72.4	*M*	A	CUNI
	*Anolis barbatus*	+	86.0	54.8	*F*	SA	CUNI
	*Anolis barbatus*	+	58.0	34.1	*M*	SA	CUNI
	*Anolis barbatus*	−	135.0	86.0	*F*	A	CUNI
	*Anolis bartschi*	−	77.0	96.3	*M*	A	CUNI
	*Anolis bartschi*	+	65.0	81.3	*M*	A	CUNI
	*Anolis bartschi*	−	58.0	91.2	*F*	A	CUNI
	*Anolis bartschi*	+	42.0	52.5	*M*	SA	CUNI
	*Anolis carolinensis*	−	65.0	91.5	*M*	A	CUNI
	*Anolis equestris*	−	115.0	67.6	*F*	A	CUNI
	*Anolis equestris*	−	160.0	84.2	*M*	A	CUNI
	*Anolis equestris*	−	135.0	79.4	*F*	A	CUNI
	*Anolis garmani*	+	95.0	86.6	*M*	A	Z. Pilsen
	*Anolis lucius*	−	48.0	80.0	*F*	A	CUNI
	*Anolis porcus*	+	122.0	75.3	*M*	A	CUNI
	*Anolis porcus*	+	69.0	40.1	*F*	SA	CUNI
	*Anolis porcus*	+	50.0	30.9	*M*	SA	CUNI
	*Anolis porcus*	+	47.0	27.3	*F*	SA	CUNI
	*Anolis roquet*	+	57.8	67.2	*M*	SA	CUNI
	*Anolis roquet*	−	71.0	82.6	*M*	A	CUNI
	*Anolis sagrei*	+	49.1	87.8	*M*	A	CUNI
	*Anolis smallwoodi*	−	160.0	84.2	*M*	A	CUNI
	*Anolis smallwoodi*	−	117.0	70.9	*F*	A	CUNI
	*Anolis vermiculatus*	−	99.0	79.5	*M*	A	CUNI
	*Anolis vermiculatus*	+	57.0	67.1	*F*	SA	CUNI
Chamaeleonidae	*Bradypodion thamnobates*	+	69.0	82.1	*M*	A	CUNI
	*Brookesia stumpffi*	+	42.0	104.6	*M*	A	Z. Zájezd
	*Brookesia thieli*	+	36.0	90.0	*F*	A	Z. Zájezd
	*Calumma brevicorne*	+	126.0	80.3	*M*	A	Z. Zájezd
	*Calumma malthe*	+	104.0	77.0	*M*	A	Z. Zájezd
	*Calumma malthe*	+	95.0	70.4	*F*	A	Z. Zájezd
	*Calumma parsonii*	+	215.0	93.1	*F*	A (>6)	Z. Zájezd
	*Calumma parsonii*	−	183.0	79.2	*F*	A	Z. Zájezd
	*Furcifer lateralis*	+	97.0	88.2	*F*	A	CUNI
	*Furcifer lateralis*	+	110.0	100.0	*F*	A	Z. Zájezd
	*Furcifer oustaleti*	+	205.0	76.0	*M*	A (>2.5)	Z. Prague
	*Furcifer oustaleti*	+	225.0	83.4	*M*	A (2)	Z. Prague
	*Furcifer oustaleti*	+	215.0	79.7	*M*	A (>2)	Z. Prague
	*Furcifer oustaleti*	+	175.0	79.3	*F*	A (>2)	Z. Prague
	*Furcifer oustaleti*	+	170.0	77.0	*F*	A	Z. Prague
	*Furcifer oustaleti*	−	232.0	86.0	*M*	A	CUNI
	*Furcifer oustaleti*	+	198.0	89.7	*F*	A	Z. Pilsen
	*Furcifer oustaleti*	±	192.0	87.0	*F*	A	Z. Zájezd
	*Furcifer pardalis*	+	175.0	70.0	*M*	A	Z. Zájezd
	*Furcifer pardalis*	+	179.0	71.6	*M*	A (>4.5)	CUNI
	*Furcifer verrucosus*	+	120.0	105.9	*F*	A	Z. Zájezd
	*Chamaeleo calyptratus*	+	157.0	52.3	*M*	A	CUNI
	*Chamaeleo calyptratus*	−	205.1	68.4	*M*	A	CUNI
	*Chamaeleo calyptratus*	−	207.0	69.0	*M*	A (4)	CUNI
	*Chamaeleo calyptratus*	±	200.9	67.0	*M*	A	CUNI
	*Chamaeleo dilepis*	+	85.0	56.7	*M*	A	Z. Zájezd
	*Chamaeleo dilepis*	+	143.0	95.3	*F*	A	Z. Zájezd
	*Chamaeleo dilepis*	+	91.0	60.7	*F*	A	Z. Zájezd
	*Chamaeleo dilepis*	+	60.0	40.0	*F*	A	Z. Zájezd
	*Kinyongia fischeri*	+	95.0	72.5	*M*	A	Z. Zájezd
	*Rieppeleon brevicaudatus*	+	44.0	58.7	*F*	A	CUNI
	*Trioceros deremensis*	+	120.0	72.7	*M*	A	Z. Zájezd
	*Trioceros deremensis*	+	120.0	75.9	*F*	A	Z. Zájezd
	*Trioceros ellioti*	+	72.0	78.3	*M*	A	Z. Zájezd
	*Trioceros hoehnelii*	+	83.0	83.0	*F*	A (3)	Z. Zájezd
	*Trioceros jacksonii*	+	104.0	78.8	*F*	A	Z. Zájezd
	*Trioceros jacksonii*	+	82.0	66.7	*M*	A	Z. Zájezd
	*Trioceros johnstoni*	+	106.0	78.5	*F*	A	Z. Zájezd
	*Trioceros melleri*	+	190.0	69.6	*F*	A	Z. Zájezd
	*Trioceros melleri*	+	257.8	89.5	*M*	A	CUNI
	*Trioceros montium*	+	101.0	109.0	*M*	A (2)	Z. Prague
	*Trioceros montium*	+	95.0	114.5	*F*	A (2)	Z. Prague
	*Trioceros montium*	+	69.0	83.1	*F*	A	Z. Prague
	*Trioceros rudis*	+	70.0	94.6	*M*	A	Z. Zájezd
	*Trioceros rudis*	+	80.0	108.1	*M*	A	Z. Zájezd
	*Trioceros rudis*	+	67.0	90.5	*F*	A	Z. Zájezd
Iguanidae	*Cyclura nubila*	−	450.0	121.6	*F*	A (21)	NMP
	*Cyclura nubila*	+	304.0	82.2	*F*	A	Z. Dubeč
	*Cyclura nubila*	+	412.1	79.3	*M*	A	CUNI
	*Dipsosaurus dorsalis*	−	114.0	80.3	*F*	A	CUNI
	*Iguana iguana*	+	450.0	118.4	*M*	A	NMP
	*Iguana iguana*	−	420.0	110.5	*M*	A (>23)	Z. Prague
	*Sauromalus ater*	−	175.0	83.3	*M*	A (>6)	Z. Prague
	*Iguana iguana*	+	410.0	107.9	*M*	A	CUNI
	*Sauromalus ater*	−	195.0	92.9	*M*	A (>8)	Z. Prague
	*Sauromalus ater*	−	180.0	90.0	*F*	A (>10)	Z. Prague
	*Sauromalus ater*	+	138.0	69.0	*F*	A	Z. Zájezd
Leiocephalidae	*Leiocephalus carinatus*	−	122.0	91.6	*M*	A	Z. Dubeč
	*Leiocephalus carinatus*	−	123.0	92.3	*M*	A	Z. Dubeč
	*Leiocephalus carinatus*	−	85.0	73.4	*F*	A	CUNI
	*Leiocephalus carinatus*	−	101.0	75.8	*F*	A	CUNI
	*Leiocephalus personatus*	−	74.0	86.0	*M*	A	CUNI
	*Leiocephalus personatus*	−	64.5	75.0	*M*	A	CUNI
	*Leiocephalus schreibersi*	+	88.0	91.7	*M*	A	CUNI
Opluridae	*Oplurus cuvieri*	±	131.5	85.5	*M*	A	CUNI
	*Oplurus fierinensis*	−	92.0	74.8	*M*	A	Z. Pilsen
	*Oplurus grandidieri*	−	118.0	84.8	*F*	A	Z. Pilsen
Phrynosomatidae	*Sceloporus malachiticus*	−	89.9	105.8	*F*	A	CUNI
Tropiduridae	*Tropidurus hispidus*	+	68.0	60.0	*F*	SA	CUNI
	*Tropidurus hispidus*	+	72.0	63.5	*F*	SA	CUNI
	*Tropidurus hispidus*	−	78.0	68.8	*F*	A	CUNI

Growth plate cartilage (GPC) presence (+), absence (−), and process of degradation (±), Snout-Vent Length (SVL) in millimetres, SVL_rel_ is relative SVL (in % of maximal SVL from the literature; for references see SI8), Sex and Age in years, where known.

Abbreviations: Male (M), Female (F), Adult (A), Subadult (SA), Charles University (CUNI), National Museum (NMP), Zoo (Z), Private Breeders (PB).

Catalogue numbers of specimens from National Museum, Prague: *Physignathus cocincinus* (NMP-P6V 75130); *Intellagama lesueurii* (NMP-P6j-29/96); *Iguana iguana* (NMP-P6V 71313).

**Figure 3:**
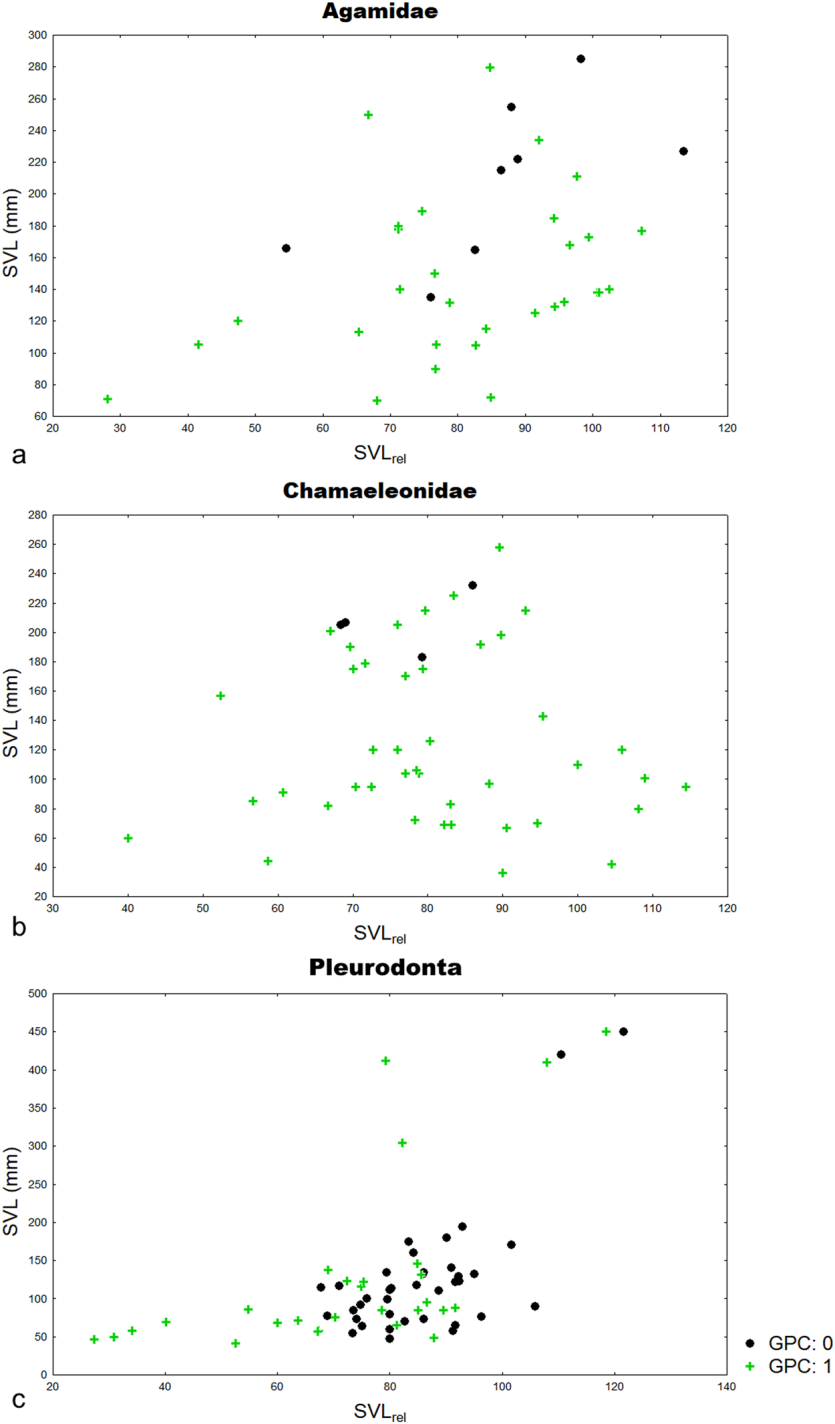
Relationship between growth plate cartilage state and body size. The presence (+GPC:1) and absence (• GPC:0) of growth plate cartilage (GPC) in agamas (**a**), chameleons (**b**), and Pleurodonta (**c**) plotted with respect to body size (SVL) and size relative to maximum SVL reported in the literature (SVL_rel_). This allows to check for the relationship between the state of GPC and body size (SVL) of the studied species, as well as between the state of GPC and the percent of attained maximal body size (SVL_rel_ in %).

We detected the presence of the growth plate cartilage in fully-grown individuals of the Acrodonta clade (Fig. 3a,b). GPC was present in almost all examined chameleons (42 individuals). Many of these animals were adults that have already reached a body size close to the upper limit reported for the species. In an old female of *Calumma parsonii*, we captured the process of GPC degradation (SI1). The only examined chameleons with completely absent GPCs were two old *Chamaeleo calyptratus* (Fig. 2b), one *Furcifer oustaleti*, and one *Calumma parsonii*. A similar pattern was found in agamid species; GPC was present in 30 individuals. Most of these animals were mature and probably fully-grown. Nevertheless, we did not find GPCs in two extremely old males of *Uromastyx loricatus* (more than 28 and 30 years old) as well as in three large-bodied agamas (a male and two females of *Physignathus cocincinus* and a male of *Intellagama lesueurii*, which were also old animals kept for many years in Prague zoo). GPC was absent also in common bearded dragon (*Pogona barbata*) and one small-bodied common butterfly lizard (*Leiolepis belliana*).

We found a completely different pattern in Pleurodonta (Iguanidae and related families), where GPC was absent in most of the adult fully-grown specimens. This GPC degradation was most apparent in anole lizards (Dactyloidae). All small-bodied anoles arrest body growth via GPC degradation early in ontogeny. In large-bodied species of anoles, we detected GPC in 4 (two *Anolis baracoe*, one *A. garmani*, one *A. porcus*) of 10 examined adults (>2/3 maximum body size). The analysis of GPC in the rest of “iguanas” revealed the absence of GPC in most of the species. We assume that in large-bodied species, similar to the pattern in monitor lizards, GPC is present for a longer time to allow growth to a bigger size. Nevertheless, in extremely old specimens kept in Prague zoo, growth is irreversibly arrested, and the GPC is completely missing (a male of *Iguana iguana* more than 23 years old, a female of *Cyclura nubila* more than 21 years old).

The disappearance of GPC in adulthood is probably coupled with a phylogenetic relationship. Acrodonta continue skeletal growth through most of their life. GPC is present even in fully-grown specimens (Fig. 3a,b), but is missing in very old/senescent individuals. In Pleurodonta, body growth arrests apparently earlier than in Acrodonta (Fig. 3c). In large-bodied members of Iguanidae, GPCs persist to adulthood and disappear just in senescent individuals.

To perform formal tests, we employed generalized linear models with a binomial distribution and phylogenetic generalized linear mixed model for binary data. The results of both models revealed a strong effect of the clade (Acrodonta versus Pleurodonta) and body size on the persistence of GPC (Table 2, Fig. 1).

**Table 2:** GLM and PGLMM model of the relationship between the growth plate cartilage persistence, clade and body size.

	Df	Deviance	Df	Resid. Deviance	p-value GLM	p-value PGLMM
Clade	1	21.28	65	71.23	<0.001	<0.001
SVL	1	11.342	64	59.89	<0.001	<0.01

The generalized linear model exploring the relationship between the growth plate cartilage (GPC) presence/absence and two explanatory variables (Clade coded as Acrodonta versus Pleurodonta and SVL-Snout-Vent Length). Interaction was not significant; binomial distribution, Chi test. The phylogenetic generalized linear mixed model for binary data (PGLMM) revealed similar results, only p-values are presented.

GPC persistence in fully grown individuals of agamids and chameleons can be caused by reduced longevity limiting the time window available for the GPC degradation process. To test this hypothesis, we explored the longevities and their relationship with body size based on large-scale comparative data^34^. Small to medium-bodied species of chameleons are typically short living. It is possible that the mortality is so high that there is not enough time for GPC degradation. On the other hand, longevities recorded in agamids do not support this explanation. There are plenty of agamid species regularly attaining longevities permitting enough time for GPC resorption. Moreover, in anole lizards which are apparently short living, the resorption of GPC is not a problem. PGLS model revealed a positive relationship between log-transformed longevity and body size in agamid (slope = 0.308, SE = 0.05, P < 0.001) and pleurodont (slope = 0.422, SE = 0.09, P < 0.001) lizards. In contrast, this relationship (slope = 0.170) was not significant in chameleons (P = 0.275). Thus, the longevity of agamids with putatively indeterminate body growth does not differ from that reported in pleurodonts which are determinate growers. This suggests that GPC degradation timing is more important than the available lifespan.

## Discussion

In our comparative study of epiphyseal growth plates, we found surprising disparity in the ability to grow throughout the lifespan within the Iguania clade. In Acrodonta, the growth plate cartilage (GPC) was present in most of the examined specimens of chameleons and agamas. The rare disappearance of GPC in senescent individuals is most probably connected with the gradual depletion of chondrocytic progenitor cells in the resting zone. This sharply contrasts with the pattern we found in Pleurodonta. Except for a few large-bodied species, GPC was resorbed in the majority of adult specimens. This suggests that pleurodonts typically resorb GPC and irreversibly arrest body growth in early adulthood. Thus, we can clearly reject the hypothesis that whole Iguania exhibit indeterminate growth.

Chameleons are a uniform and morphologically highly derived family with numerous specialized adaptations connected with their arboreal life style^35^. Members of this family covering dwarf as well as medium-sized species are early maturing and short-living lizards (e.g., the extremely short lifespan in *Furcifer labordi*^36^) with large clutch size (even up to 50 eggs) laid usually once or twice per year. In our study, we found preserved GPC in most of the studied chameleons, which points to their potential ability to grow throughout the life. The presence of GPC even in small-bodied species and the absence of a relationship between the preservation of GPC and body size (Fig. 3b) is in contrast to our findings in monitor lizards, where GPC was absent in all small-bodied species. It may be advantageous to preserve GPC in adulthood and keep the ability to grow, because larger body size is associated with higher survival probability and reproduction success (for a theoretical model see^37^). Moreover, chameleons exhibit pronounced sexual size dimorphism^38,39^. Sexual selection in males^40^ as well as fecundity selection in females^38^ appear as candidate ultimate mechanisms responsible for the apparent size and shape dimorphism in this family. Moreover, as many species of chameleons are seasonal^35,36^, the presence of GPC throughout the lifespan allows to alternate a period of dormancy with an active growth period, and supports the theoretical framework of Ejsmond^41,42^ as well as the scarce long-term research in other reptiles with indeterminate growth^37^. Our results suggest no relationship of GPC degradation timing with sex (most of the studied animals were kept in pairs, see Table 1), which challenges the cost of reproduction hypothesis^43–45^. The only exceptions with arrested growth were an old male of *Furcifer oustaleti*, *Calumma parsonii* and two old males of *Chamaeleo calyptratus*. Nevertheless, the *Furcifer oustaleti* specimen had a metabolic bone disease manifested by bone decalcification, which is typical for old captive-bred chameleons^46^. The *Chamaeleo calyptratus* specimens were healthy with no signs of a metabolic bone disease. In this case, GPC degradation was probably connected with high age (one individual was 4 years old). *Chamaeleo calyptratus* is short-living^46^, males have a longer lifespan in captivity (3–5 years) than females (2–3 years). Despite these exceptional nearly senescent individuals, we assume that members of the family Chamaeleonidae preserved the GPC (and thus at least a theoretical growth ability) throughout their short life, because they usually die long before the eventual GPC resorption.

We found a similar distribution in agamid species, which preserved GPC throughout their life, as like in chameleons. This group with a wide spectrum of body sizes (from the smallest genus of *Draco* to the large-bodied *Intellagama*) comprises rather short-living (e.g., *Draco volans*^47^, *Ctenophorus isolepis*^48^, *C. maculosus*^49^) or even annual species (e.g., *C. fordi*^50^, *C. nuchalis*^51^). There are exceptions, as some medium and large-bodied species of the genus *Uromastyx*^52^, *Physignathus*^53^ and *Hydrosaurus*^54^, are long-lived (>33 years old). The clutch is medium-sized (mean = 8.3 eggs) and appears once or twice per season (according to Scharf’s review^34^). Our results revealed that GPC is present even in individuals which have already reached the maximum of the species-specific body size, and GPC presence is not dependent on the species-specific body size (GPC is present even in small-bodied species). GPC degradation appeared just in very old individuals, which were kept in Prague zoo and by private breeders for many years (*Physignathus cocincinus*, *Intellagama lesueurii* and *Uromastyx loricatus*) and two specimens of other species of unknown age. Two examined specimens of Iraqi spiny-tailed lizard (*U. loricatus*) were more than 28 years old. We assume that in those large-bodied individuals, GPC is present for a longer time to attain a larger body size, nevertheless, in such almost senescent individuals, there was enough time for its resorption through gradual depletion of chondrocytic progenitor cells in the resting zone of GPC. We found a similar pattern in the large-bodied senescent mangrove-dwelling monitor lizard (*Varanus indicus*^22^).

Surprisingly, we found a completely opposite pattern in the closely related Pleurodonta, where GPC disappears early in ontogeny (even in animals which reached only 70% of maximal SVL, Fig. 3c). This pattern was universal for all studied “iguanid” families (Corytophanidae, Crotaphytidae, Dactyloidae, Iguanidae, Leiocephalidae, Opluridae, Phrynosomatidae, Tropiduridae), but in anole lizards was most apparent. “Iguanas”, as a convergent lineage of agamas, feature a broad body size spectrum and various ecological strategies. The mean longevity is comparable in Acrodonta and Pleurodonta, but extreme longevities were recorded in Iguanidae (e.g., 60 years in *Conolophus pallidus* and *C. subcristatus*^55^; 54 years in *Cyclura nubila*^56^ and 40 years in *C. cychlura*^57^). The mean clutch size is a bit smaller in “iguanas” (7.1 eggs according to Scharf’s review^34^) and reaches the extreme in anoles, which produce invariant clutches (only one egg per clutch^58,59^).

In most of the examined anoles, GPC was not present, and they arrested growth irreversibly early in ontogeny (Fig. 3c). The exceptions were found in four specimens of large-bodied species (*Anolis baracoae*, *A. garmani* and *A. porcus*, which were almost fully-grown, SVL_rel_ = 75–90%). We analysed 32 individuals (13 species), and both sexes were usually available. Most of the animals were from one private breeder (V. Z.), regularly bred and were kept under standard common garden conditions. We predicted the difference in timing of GPC resorption according to the sex of the examined animal. Male anoles are territorial^60–62^ and it should be advantageous to them to have the opportunity to invest in body growth for a longer period of ontogeny (i.e., preserve GPC to maintain the growth ability). On the other hand, the cost of reproduction is high in anole females and the reduction to one egg per reproductive event is believed to reduce the female reproductive burden^63^ (but see^64^). Even though there is only one egg per clutch, the number of broods per year can be very high (e.g., an extreme case of 25 egg layings per year in *A. roquet*^56^). Consecutive ovulation and egg laying bring elevated levels of progesterone and derivatives of oestrogen^65,66^. It was experimentally verified that elevated levels of female gonadal hormones (derivatives of oestrogen^67,68^ and progesterone^69^) accelerate growth plate senescence. This proximate mechanism should enhance growth plate resorption and arrest skeletal growth in females earlier than in males. But we did not observe any sexual dimorphism in the timing of GPC resorption in our dataset.

We found remarkable results in chuckwallas (*Sauromalus*, Iguanidae) and spiny-tailed lizards (*Uromastyx*, Agamidae). These desert herbivorous lizards share many life-history parameters and are considered as ecologically convergent species. But GPC is present in spiny-tailed lizards for a longer time in ontogeny than in chuckwallas. We don’t know the exact age of some of the examined spiny-tailed lizards as these specimens were maintained by the zoo after their confiscation from illegal trade in 2008. Some of them were juveniles, most of them subadults. Thus, at the time of our analysis, they were more than 10 years old and still possessed GPC. The only exceptions found were the two senescent individuals of *Uromastyx loricatus* mentioned above, which were more than 28 years old. In this case, GPC was fully resorbed. In *Sauromalus*, GPC was completely resorbed in two males, which were older than 6 and 8 years, respectively, and in a 10 years old female. This is another piece of evidence that the timing of GPC resorption differs in acrodonts and pleurodonts and is not primarily connected with their ecology.

The great difference we uncovered in the timing of GPC degradation between Acrodonta and Pleurodonta (Fig. 1) was unexpected. These sister clades diversified in parallel in the New and Old world, respectively, forming plenty of ecologically and morphologically corresponding forms. The absence of GPC in adults of the examined pleurodonts clearly suggests an irreversible arrest of growth in this clade. In contrast, GPC preservation in the vast majority of adult acrodonts provides less clear evidence for indeterminate growth. Putatively, the results may be biased by the composition of the examined material, namely by the precise stage of ontogeny, body size, age and their interactions. As the growth parameters may exhibit great interindividual variation^70,71^, it is difficult to control it. Nevertheless, there is a study strongly supporting indeterminate growth in an agamid lizard. Kumaş & Ayaz^72^ studied longevity and long bone development in four wild populations of Roughtail Rock Agama *(Stellagama stellio)* in Turkey. Analysis of LAGs (Lines of Arrested Growth) revealed that these agamas grow throughout their life although the increments are much smaller in older age. Transverse cross sections of the epiphysis in different age groups revealed gradual resorption of GPC in the femur. The maximum age detected for both sexes was 7 years, and even in such old individuals, GPC was thinner but still present^72^. The finding that the preservation of GPC to adulthood is accompanied by continuation of body growth is especially remarkable. This provides independent evidence for the interpretation of our data.

Our results suggest that there are at least two modes of GPC resorption timing in squamate reptiles. The first one comprises early timing of GPC resorption. Although the final body size is typically attained after a certain delay, it is associated with the timing of sexual maturation (for a theoretical background see^73^). Such evidence was found in Pleurodonta as well as in small species of monitor lizards. It is also supported by the ontogenetic study of body growth in the Madagascar ground gecko (*Paroedura pictus*), which revealed an abrupt process of GPC degradation^74^. The second mode of GPC resorption timing is completely different. The resorption is either considerably postponed or it is not even realised. We found this pattern in Acrodonta and large-bodied monitor lizards. It may be labelled as indeterminate growth; however, it depends on the applied definition of this term^3,10,25,75^.

Our results support a large-scale comparative study of lepidosaurs, which was published recently^34^. Scharf and his colleagues corroborated the key prediction from life-history theory and suggested that: “reproducing more slowly and at older ages, being herbivorous and, plausibly, lowering metabolism, result in increased longevity.” Nevertheless, body size explains far less of the variation in longevity than it does in mammals and birds, which is a surprising finding when squamates are considered as indeterminate growers. It is obvious that body growth is more plastic in squamate reptiles, and diverse ecology and life-history strategies affect it tremendously^76–78^.

We discuss our findings in a wider phylogenetic context. Our previous study of the Anguimorpha clade revealed determinate body growth in small and medium-bodied lizards (*Heloderma, Shinisaurus, Varanus*), while large-bodied monitor lizards were scored as indeterminate growers^22^. The visualisation of GPC in the Iguania clade supports these results, even though GPC presence/absence was not connected with body size so tightly. We found common absence of GPC also in other adult specimens of Squamata (Gekkota, Scincomorpha and Lacertoidea; unpublished results). Moreover, analysis of bone rings and growth data suggest determinate growth in tuataras (*Sphenodon punctatus*) as well^14,79^. Thus, the ancestor of Lepidosauria was most probably a determinate grower.

In conclusion, we were able to analyse a large number of femurs by micro-CT. The resolution is high (even 2 µm, according to the femur size) and make it possible to employ micro-CT in studies investigating the growth plate cartilage and the dynamics of its degradation. We found surprising disparity of GPC presence/absence in the Iguania clade. In Acrodonta, GPC is present nearly throughout the life and disappears in very old and senescent animals. Thus, growth is not completely blocked in agamas and chameleons (via GPC resorption) and they can be considered as indeterminate growers. On the other hand, Pleurodonta arrest skeletal growth earlier in ontogeny (GPC irreversibly disappears) and can be regarded as determinate growers. We interpret the uncovered GPC disparity as a unique switch in timing of growth arrest signalization leading to a postponed or even cancelled process of GPC resorption. Taken together with the evidence of determinate body growth in other squamate lineages and tuataras, we interpret our results as a significant challenge to the universality of indeterminate growth in Lepidosauria.

## Material and Methods

### Data collection

We analysed 70 species/subspecies of lizards from the Iguania clade (for the list see Table 1) which were selected to capture most of the diversity of Iguania, comprising families Agamidae (21 species/37 individuals), Corytophanidae (4/6), Crotaphytidae (1/3), Dactyloidae (13/33), Chamaeleonidae (22/46), Iguanidae (4/11), Leiocephalidae (3/7), Opluridae (3/3), Phrynosomatidae (1/1) and Tropiduridae (1/3). We aimed to cover the spectrum of body sizes, longevities, ecologies, and life strategies. Samples were collected in zoological gardens, collections of the Department of Zoology and museum collections from animals died of natural death. In addition, we used cadavers of lizards from another morphological study running at the Department of Zoology (34 individuals).

We were primarily interested in very large and/or old adult individuals approaching their maximal specific age and body size. We included also a few specimens that were younger and apparently still growing as controls. The snout-vent length (SVL) of each specimen was measured to the nearest 0.1 mm and expressed as an absolute (SVL) and relative (SVL_rel_) value. The latter represents a percent ratio of SVL of the examined specimen relative to the maximum SVL reported in the literature for the particular species and sex (the data concerning SVL are summarized in Table 1, SVL_max_ and references in SI8). The maximal SVLs from the literature are often overestimated. In a plot of regressed SVL to SVL_rel_ and the growth plate cartilage presence/absence as a categorical variable, it is possible to show a relationship between those variables in various species/clades and the timing of growth plate degradation. In some cases, our specimens are the largest or oldest ones ever reported, and thus, their relative size exceeds 100%. These represent additional evidence that we succeeded to include specimens reaching the upper limits of body size and/or age attainable by the examined species. Nevertheless, it is important to note that maximal body size is usually larger in captive-bred animals than in wild populations (e.g., compare body size in captive bred vs. wild *Varanus indicus*^70,80^).

The femur was used for analysis as it is the largest long bone in the body. The bone was dissected and mechanically purified. The proximal part of the bone was analysed. The presented measurements were carried out at micro-CT laboratory of the Institute of Experimental and Applied Physics (IEAP), Czech Technical University in Prague, and at Specialized Laboratory of Experimental Imaging (joint laboratory of the Third Faculty of Medicine, Charles University, IEAP and Faculty of Biomedical Engineering, Czech Technical University in Prague). While the Specialized Laboratory of Experimental Imaging is equipped with a Bruker SkyScan 1275 micro-CT scanner and a customized micro-CT system designed for small animal imaging^81^ the IEAP laboratory operates two in-house developed micro-CT systems utilizing large-area photon counting detectors based on Timepix technology^82^. The SkyScan 1275 was used for scanning of large samples as it is equipped with a highly efficient and fast CMOS flatpanel detector, while the custom systems at laboratory of IEAP were utilized for smaller samples since higher resolution and higher contrast-to-noise ratio could be achieved using these set-ups^83,84^.

The scan parameters were adjusted for each sample individually according to its size and attenuation properties. Generally, the samples were scanned in cone-beam geometry with angle step 0.2–0.4 degree and using 40–60 kVp unfiltered tungsten spectrum. The acquired micro-CT data were reconstructed using filtered back projection algorithm via NRecon software or Volex reconstruction engine (courtesy of Fraunhofer-Allianz Vision, Germany) in the case of SkyScan 1275 or custom set-up respectively. The voxe-size of the reconstructed slices was within the range of 4–13 µm. The data analysis was carried out using Fiji^85^(video creation) and CTVox^86^(data survey and figure creation).

We evaluated the epiphyseal senescence and ossification status (the presence or absence of the growth plate) blindly by two independent observers. The criteria for senescence included a diminished chondrocytic area of the growth plate between the epiphysis and the metaphysis, and the absence of the suture between the metaphysis and epiphysis. In addition, the inner structure of the epiphysis was assessed in detail using 3D visualizations made from micro-CT scans. Juveniles and subadults possess a more dense epiphyseal structure lacking the typical trabecular architecture. Later, the endochondral ossification process in secondary ossification centres is completed and typical trabecular bone architecture appears in the epiphysis of older animals. Finally, the growth plate disappears, and the trabecular bone architecture fills up the space of metaphysis.

We complemented our analysis with additional data from literature concerning the maximal body size and longevity for the Iguania clade. Most data come from Scharf’s large-scale comparative study^34^, which analysed the relationship of longevity, environmental characteristics and life-history traits.

### Statistical analyses

We employed a generalized linear model (GLM) to explore the relationship between GPC presence/absence (binary coded) and two explanatory variables (body size expressed as snout-vent length and clade coded as Acrodonta versus Pleurodonta) and their interaction in R^87^. Since species cannot be considered as independent data points^88^, we run analysis accounting for the effect of phylogeny as well. We used phylogenetic generalized linear mixed model for binary data (PGLMM)^89,90^ implemented in R package ‘ape’^91^. We adopted a time-calibrated phylogeny of squamata^33^ even though the relationships inside Pleurodonta remain uncertain. Because the pattern of GPC degradation is homogenous within pleurodonts (GPC usually resorbed), incompletely resolved pleurodont phylogeny does not significantly affect results of the analysis. In both types of models, we used only one individual per species and included only mature individuals (with SVL_rel_ > 75%)^73^ or those with resorbed GPC (final dataset contained 68 species). For visualisation of different timing in Acrodonta and Pleurodonta, we plotted the relationship between SVL and SVL_rel_ with GPC presence/absence as a categorical variable in STATISTICA, version 6^92^ (all specimens were included). The Mesquite programme (version 3.51) was employed for the ancestral state reconstruction of GPC on a phylogenetic tree^93^ (the same dataset as for GLM models). Final circular cladogram was visualized in Dendroscope 3^94^. We scored the GPC binary as absent vs present (GPC clearly visible as well as nearly resorbed, but still a little bit present) in all analyses. Statistical models concerning longevity were performed using PGLS method^95,96^ implemented in R packages ‘ape’^91^ and ‘nlme’^97^. The log-transformed data were adopted from Scharf ^34^, phylogeny and branch lengths from Zheng and Wiens^33^.

## Supplementary information

SI1

SI2

SI3

SI4

SI5

SI6

SI7

SI8

## Data Availability

All data generated or analysed during this study are included in this published article (and its Supplementary Information files).
