# Supplementary material for: Universality of indeterminate growth in lizards rejected: the micro-CT reveals contrasting timing of growth cartilage persistence in iguanas, agamas, and chameleons
Source: Sci Rep. 2019 Dec 12;9:18913. doi: 10.1038/s41598-019-54573-5 (PMC6908584; doi:10.1038/s41598-019-54573-5)

Universality of indeterminate growth in lizards rejected: the micro-CT reveals contrasting timing of growth cartilage persistence in iguanas, agamas, and chameleons

Petra Frýdlová[1,3], Jana Mrzílková[2,3], Martin Šeremeta[2,3], Jan Křemen[2,3], Jan Dudák[2,4], Jan Žemlička[2,4], Pavel Němec[1], Petr Velenský[5], Jiří Moravec[6], Daniel Koleška[7], Veronika Zahradníčková[1], Tomáš Jirásek[8], Petr Kodym[9], Daniel Frynta[1*], Petr Zach[2,3]

SI 1. Visualization of growth plate cartilage (GPC) degradation by micro-CT. Frontal cross-section of the proximal part of the femur in adult old *Calumma parsonii*. The epiphyseal growth plate is in the process of gradual degradation. Abbreviations: Epiphysis (Epi), Metaphysis (Met), Diaphysis (Dia), Suture (Sut), Epiphyseal growth plate (asterisk). Note the incomplete suture in the metaphyseal region and partly resorbed GPC. Bar 20 μm.

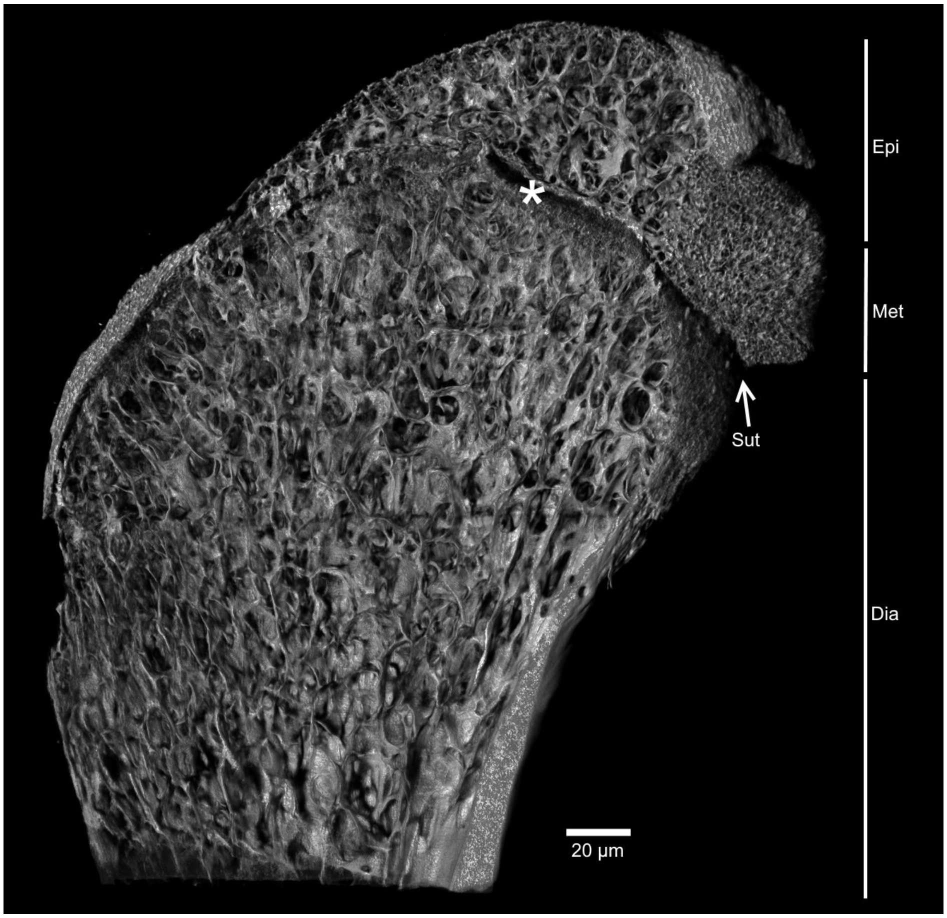

Supplement: Supplementary file 1 — SI1 [file 41598_2019_54573_MOESM1_ESM.pdf]
